# Cloud micro- and macrophysical properties from ground-based remote sensing during the MOSAiC drift experiment

**DOI:** 10.1038/s41597-024-03325-w

**Published:** 2024-05-16

**Authors:** Hannes J. Griesche, Patric Seifert, Ronny Engelmann, Martin Radenz, Julian Hofer, Dietrich Althausen, Andreas Walbröl, Carola Barrientos-Velasco, Holger Baars, Sandro Dahlke, Simo Tukiainen, Andreas Macke

**Affiliations:** 1 Leibniz Institute for Tropospheric Research, Remote Sensing of Atmospheric Processes, Leipzig, 04318 Germany; 2 University of Cologne, Institute for Geophysics and Meteorology, Cologne, 50969 Germany; 3 Alfred-Wegener-Institute, Atmospheric Physics, Potsdam, 14473 Germany; 4 Finnish Meteorological Institute, Atmospheric Composition Research Unit, 00101 Helsinki, Finland

**Keywords:** Atmospheric dynamics, Attribution, Research data

## Abstract

In the framework of the Multidisciplinary drifting Observatory for the Study of Arctic Climate Polarstern expedition, the Leibniz Institute for Tropospheric Research, Leipzig, Germany, operated the shipborne OCEANET-Atmosphere facility for cloud and aerosol observations throughout the whole year. OCEANET-Atmosphere comprises, amongst others, a multiwavelength Raman lidar, a microwave radiometer, and an optical disdrometer. A cloud radar was operated aboard Polarstern by the US Atmospheric Radiation Measurement program. These measurements were processed by applying the so-called Cloudnet methodology to derive cloud properties. To gain a comprehensive view of the clouds, lidar and cloud radar capabilities for low- and high-altitude observations were combined. Cloudnet offers a variety of products with a spatiotemporal resolution of 30 s and 30 m, such as the target classification, and liquid and ice microphysical properties. Additionally, a lidar-based low-level stratus retrieval was applied for cloud detection below the lowest range gate of the cloud radar. Based on the presented dataset, e.g., studies on cloud formation processes and their radiative impact, and model evaluation studies can be conducted.

## Background & Summary

Clouds play a critical role in the processes driving the accelerated surface warming in the Arctic, known as Arctic amplification. Clouds have a direct impact on the energy balance, e.g., by alternating radiative fluxes, but also via moisture and heat transport, and play a dominating role in the surface energy budget^1,2^. The cloud-feedback, which refers to variations of the cloud effect due to changes in other factors, such as the surface temperature, has been attributed a rather small role in Arctic amplification^3–5^. Yet, there is still a large intermodel spread of the cloud feedback and the sign of the cloud feedback varies in different models^6^. Despite many advances made in research on the role of clouds in Arctic amplification in recent years^7–9^ there are still some gaps in the understanding of the interactions between clouds and other feedback mechanisms, such as the ice-albedo-feedback or the lapse-rate-feedback. Additionally, the largest model uncertainty in terms of radiative forcing is attributed to the yet not quantified interaction between clouds and aerosol particles^10^. Model simulations and satellite observations show an annually averaged net warming effect of Arctic clouds at the surface of approximately 20 W m^−2^, with a strong seasonal cycle^11^. The net cloud radiative effect at the surface is strongly influenced by the cloud thermodynamic structure, and its microphysical and optical properties^12^. It is the liquid phase in the cloud that dominates the respective surface radiative effect by decreasing the downward solar radiation and increasing the downward terrestrial radiation^13^. Mixed-phase cloud formation and their micro- and macrophysical properties are subject to atmospheric thermodynamic processes and aerosol abundance^14^. So-called cloud condensation nuclei (CCN) are necessary for droplet formation, and heterogeneous ice formation at sub-zero temperatures down to about −40 °C needs the availability of ice nucleating particles (INP). An increase in CCN can enhance the cloud optical thickness and lifetime due to a higher droplet number concentration and a resulting decrease in the droplet size^15,16^. An increase in INP concentration can initiate cloud dissipation through enhanced precipitation^17^.

Remote sensing is a valuable tool to determine cloud micro- and macrophysical properties^18–21^. These observations and the derived properties can, e.g., be used for studies on cloud and precipitation formation^22–24^, aerosol-cloud-interaction processes^25–27^, or the cloud influence on the radiation budget^21,28–30^. A special feature of Arctic clouds is their longevity, which can be up to several days^23,31,32^, and the low altitude where they can occur, which is frequently below the lowest height limit of remote-sensing instruments^19^. These clouds frequently show ice precipitation^23^ and the necessary supply of ice nucleating particles is not clear^33^. Recent studies indicate that biogenic processes in the marginal ice zone play an important factor^26,34,35^. Recently, an annual cycle of the vertical distribution and abundance of aerosols in the Arctic was presented^36^. Higher concentrations of cloud-forming particles were observed during the Arctic haze season in winter time compared to summertime in the atmospheric boundary layer. In the lower free troposphere, the seasonal variations of CCN were found to be less pronounced. INP were found to be reduced in the lower free troposphere in the summer compared to winter. Cloud statistics based on remote sensing performed at the coastal sites of Utqiaġvik, Alaska, US, and Ny-Ålesund, Svalbard, revealed an annual cycle of cloud occurrence, with a maximum cloud fraction during autumn and a minimum during spring^21,37^. The liquid cloud fraction at both sites was highest during summer and lowest during winter, while ice clouds were observed more frequently during winter than during summer. One important factor, driving the ice formation in clouds is the temperature^17,38–40^. However, the interaction between the temperature dependence and other atmospheric parameters, such as the availability of cloud-active aerosol particles or turbulence, in cloud-ice formation is still subject to current research^14,34,41^. Several studies have investigated the cloud cover of the Arctic. A small but evident decreasing trend of Arctic cloud fraction has been identified between 1981 and 2012 based on satellite observations^42^. Jenkins *et al*.^43^ analyzed changes in cloud properties and atmospheric conditions derived from reanalysis and satellite data for regions with and without large sea ice loss in the Arctic from 1950–1979 to 1990–2019. They highlighted an increase of cloud fraction and liquid and ice water content over areas with strong sea ice loss around the height levels between 950–700 hPA and a decrease of cloud fraction around 1000-950 hPa during October–March. Philipp *et al*.^44^ identified a cloud-sea ice feedback mechanism for autumnal cloud cover in the Arctic using satellite observations. The authors showed that a smaller sea ice cover is leading to more low-level clouds, which results in turn again in less sea ice concentration. A shift of cloud water from the liquid to the ice phase for clouds distributed across the Arctic was identified by Lelli *et al*.^45^ based on more than 20 years of satellite observations of the spectral reflectance between 1996 and 2018. They observed a balancing of the surface albedo decrease caused by the sea ice retreat due to an increase of the top of the atmosphere atmospheric reflectivity. The authors reported evidence for a tendency of locally reduced cloud radiative forcing at the surface as a consequence of the higher cloud reflectance, especially for regions and seasons with strong sea ice reduction.

**Fig. 1:**
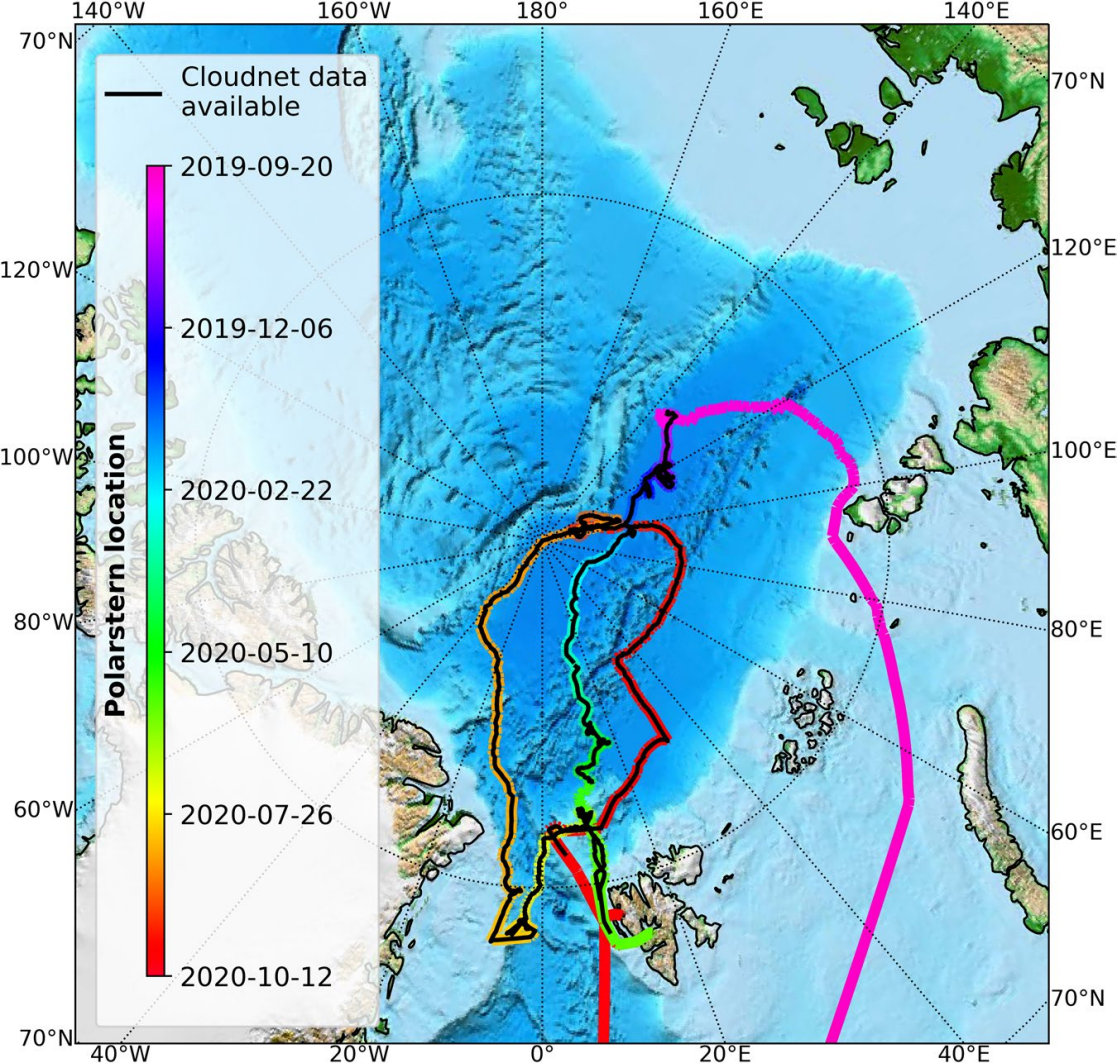
The location of Polarstern during the MOSAiC expedition is shown by the colored line. The black line highlights the part of the track where Cloudnet data are available. Map created with cartopy^121^.

To study the effects driving Arctic amplification, the German Transregional Collaborative Research Centre TRR 172, “ArctiC Amplification: Climate Relevant Atmospheric and SurfaCe Processes, and Feedback Mechanisms (AC)^3^”^9,46^ was initiated. The (AC)^3^ project conducted and participated in several Arctic field campaigns, and contributed, amongst others, to the year-long Multidisciplinary drifting Observatory for the Study of Arctic Climate (MOSAiC) expedition^47^. The MOSAiC expedition has been conducted in the high Arctic (Fig. 1) from September 2019 to October 2020 to gain a more comprehensive view of the climate-relevant processes in the Arctic. During the MOSAiC expedition, continuous measurements with the OCEANET-Atmosphere facility from the Leibniz Institute for Tropospheric Research (TROPOS), Leipzig, Germany, were performed^25^. The OCEANET-Atmosphere measurements, together with cloud radar observations from the Ka-band zenith pointing cloud radar KAZR from the US Atmospheric Radiation Measurement (ARM) program and radiosonde profiles, were used to derive the dataset of cloud micro- and macrophysical properties introduced in the following. The data were processed applying the instrument synergy approach of Cloudnet^18,48^, similar to the processing of the data from the Polarstern^49^ cruise PS106^50^ Cloudnet dataset^19^. Cloudnet offers time-height profiles of cloud macro- and microphysical properties on a continuous basis. The black line in Fig. 1 shows the part of the MOSAiC cruise during which Cloudnet data are available.

## Methods

**Fig. 2:**
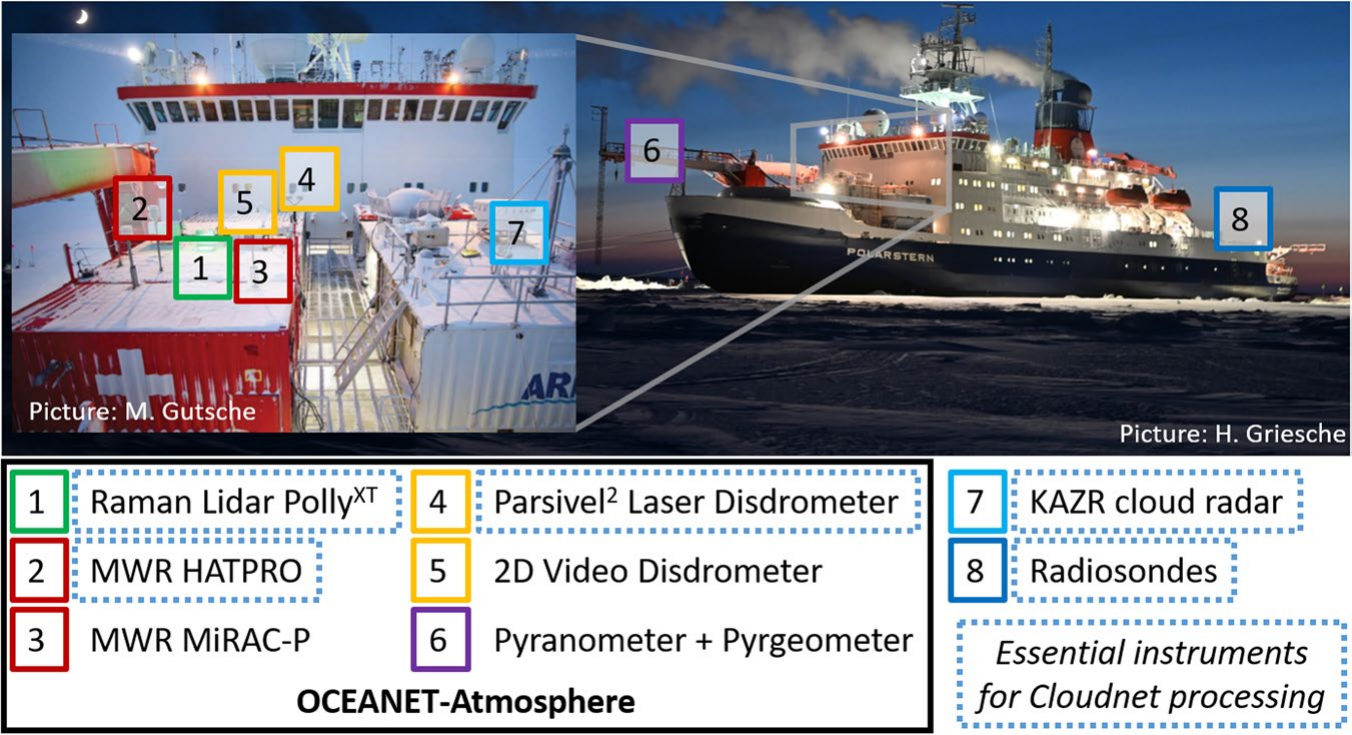
Overview of the instrument locations during the MOSAiC expedition. Instruments highlighted by a dotted blue frame were applied in the Cloudnet processing.

In this Section, first, an overview of the measurements is given. Figure 2 shows the location of the instruments that were used to derive the presented dataset. Finally, the Cloudnet retrieval and the detection of low-level stratus (LLS), clouds that were present below the lowest cloud radar height detection limit, are introduced.

### Atmospheric measurements for Cloudnet processing

During the MOSAiC expedition, several remote-sensing instruments were operated, such as those located in the OCEANET-Atmosphere facility. During MOSAiC, OCEANET-Atmosphere provided Cloudnet-relevant input datasets from lidar, microwave radiometer (MWR), and a precipitation sensor. Also, the ARM cloud radar KAZR was measuring quasi-continuously during the MOSAiC expedition. The KAZR is part of the ARM Mobile Facility 2 (AMF2). Throughout the entire measurement time, Vaisala RS41 radiosondes were launched at least every 6 hours. The radiosondes provide profiles of the atmospheric pressure, humidity, temperature, and 2-D wind vector up to a height of approximately 30 km. The measurements were provided with a temporal resolution of 1 s and the vertical resolution depends on the ascent rate (approximately 5 m s^−1^). Additionally, the ship motion was continuously recorded during the cruise. The radiosonde profiles and the ship motion data were published by the University of Cologne and the Alfred-Wegener-Institute via PANGAEA^51,52^.

**Fig. 3:**
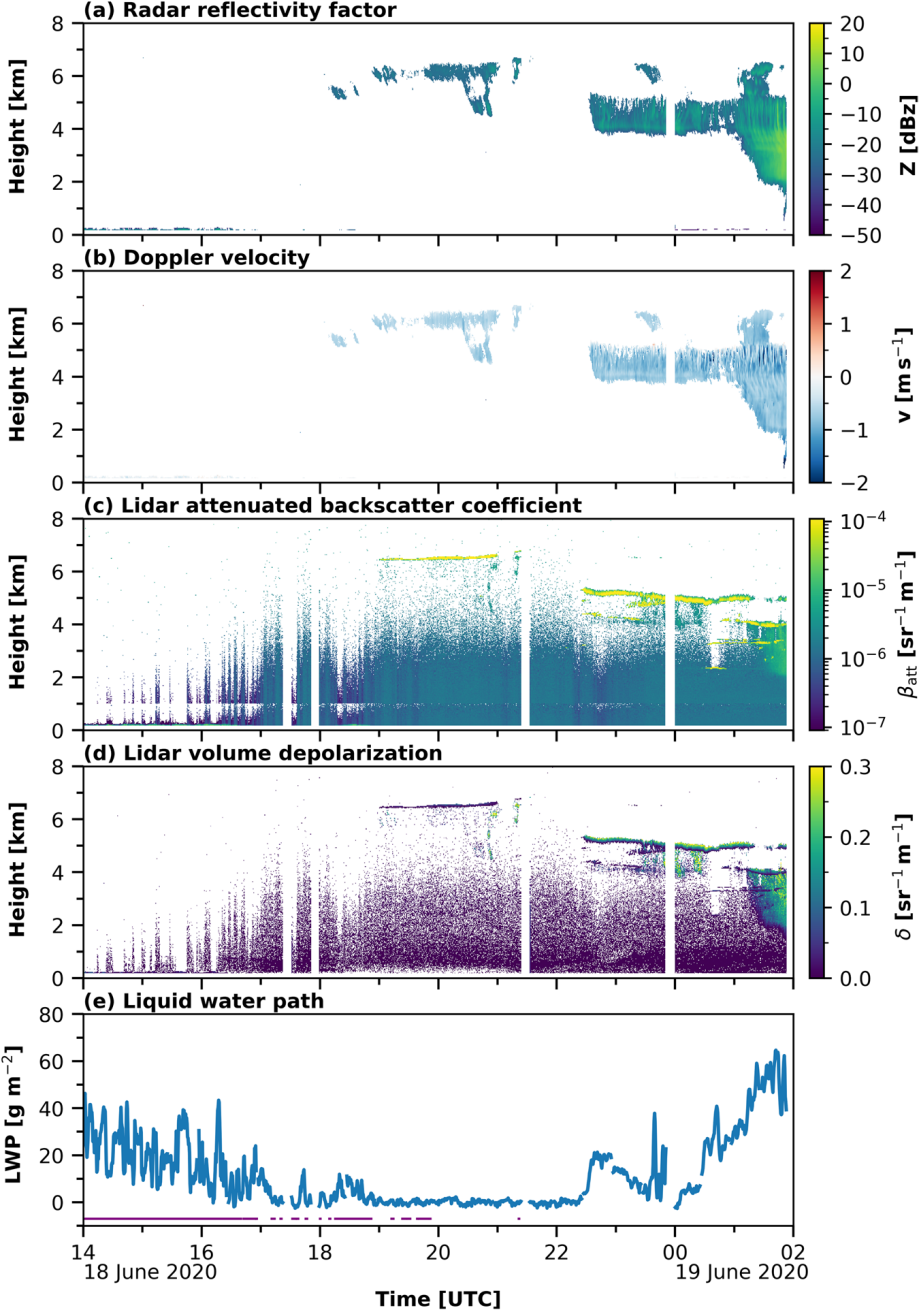
Observations applied in the Cloudnet processing from 18 June 2020 14:00 until 19 June 2020 02:00 UTC. In panel (**a**) and (**b**) the cloud radar reflectivity and Doppler velocity are presented, respectively. Panel (**c**) and (**d**) depict the lidar attenuated backscatter and volume depolarization ratio, respectively. In panel (**e**) LWP from MWR HATPRO is shown by the blue line and the presence of LLS is indicated by the purple flag.

Based on the lidar, MWR, disdrometer measurements, cloud radar observations, and the radiosonde profiles, a Cloudnet dataset for the whole drift year was derived^53–58^. The details and specifications of the relevant instruments are summarized in Table 1. In Fig. 3, the cloud radar reflectivity and Doppler velocity together with the temperature field, the lidar attenuated backscatter coefficient and volume depolarization at 532 nm wavelength, and the MWR liquid water path (LWP) together with an LLS-flag indicating clouds below the lowest cloud radar range gate, are shown for a case study from 18 June 2020 14 UTC to 19 June 2020 2 UTC. In the following, the OCEANET-Atmosphere facility and the cloud radar KAZR are introduced in more detail.

**Table 1:** Applied instruments and their specifications.

Instrument Type *Platform*	Derived Quantity	*v*: Frequency *λ*: Wavelength R: Range of Measurement P: Precision	T: Time Resolution V: Vertical resolution
Raman Lidar Polly^XT^^63^ *OCEANET*	Backscatter coefficient	*λ* = 355, 532, 1064 nm R: 0.1–20 km, 0–1 km^−1^ sr^−1^ P: 10^−5^ km^−1^ sr^−1^	T: 30 s V: 7.5 m
	Volume depolarization ratio	*λ* = 355, 532 nm R: 0.1–20 km, 0–0.5 P: 0.01	
	Particle backscatter coefficient	*λ* = 355, 532, 1064 nm R: 0.3–20 km, 0–1 km^−1^ sr^−1^ P: 10^−5^ km^−1^ sr^−1^	T: 30 min^−1^–1 hr^−1^ V: 300 m
	Particle linear depolarization ratio	*λ* = 355, 532 nm R: 0.3–20 km, 0–0.5 P: 0.01	
	Particle extinction coefficient	*λ* = 355, 532 nm R: 0.3–5 km, 0–10 km^−1^ P: 10^−2^ km^−1^	
Microwave Radiometer RPG HATPRO-G5^61,76^ *OCEANET*	Integrated water vapor (IWV)	*v* = 22.24–31.4 GHz R: 0–35 kg m^−2^ P: 0.3 kg m^−2^	T: 1 Hz
	Liquid water path (LWP)	*v* = 22.24–31.4 GHz R: 0–1 kg m^−2^ P: 0.02 kg m^−2^	
	Brightness temperature (TB)	*v* = 51.0–58.0 GHz R: 0–330 K P: 0.2–1 K	
Laser Disdrometer OTT Parsivel^2^ ^62^ *OCEANET*	Hydrometeor size distribution	*λ* = 880 nm R: 0.2–8 mm (liquid) R: 0.3–25 mm (solid) Sampling area 0.18 m × 0.03 m	T: 30 s
Pyranometer Kipp and Zonen CMP21^119^ *OCEANET*	Solar irradiance	R: 0.285–2.8 *μ*m	T: 5 s (response time)
Pygeometer Kipp and Zonen CGR4^119^ *OCEANET*	Terrestrial irradiance	R: 0.3–1.1 *μ*m	T: 18 s (response time)
Doppler cloud radar Ka-Band ARM Zenith Radar^78^ (KAZR) *AMF2*	Radar reflectivity factor	*v* = 35.5 GHz R: 0.18–18 km; -55–20 dBZ P: 2 dBZ	T: 2 s V: 30 m
	Hydrometeor vertical velocity	*v* = 35.5 GHz R: 0.18–18 km; -6–6 m s^−1^ P: 0.08 m s^−1^	
	Spectral width	*v* = 35.5 GHz R: 0.18–18 km; 0.03–3 m s^−1^ P: 0.08 m s^−1^	
Radiosonde RS41^120^ *Polarstern*	Atmospheric pressure	P: 1 hPa (>100 hPa), R: surface to 3 hPa	T: 1 s (launch at least every 6 hours) V: 5 m at 5 m s^−1^ ascend speed
	Atmospheric humidity	P: 4% R: 0–100%	
	Atmospheric temperature	P: 0.3 °C (<16 km) R: −90–60 °C	
	Atmospheric 2-D wind vector	P: 2° (>3 m s^−1^) P: 0.15 m s^−1^

#### OCEANET-Atmosphere facility

The marine mobile measurement facility OCEANET-Atmosphere (hereafter referred to as OCEANET) is operated by TROPOS since 2008 in order to perform atmospheric profiling from research vessels such as Polarstern and Meteor^19,59,60^. OCEANET is equipped by default with a multiwavelength polarization Raman lidar Polly^XT^ and a 14-channel MWR HATPRO Generation 5^61^. Also, an additional MWR MIRAC-P of type LHUMPRO, an OTT laser disdrometer Parsivel^2^ ^62^, a 2-D video disdrometer, and a pyranometer and a pyrgeometer were installed and operated within OCEANET during MOSAiC.

The lidar system Polly^XT^ provides profiles of the attenuated backscatter coefficient at 355, 532, and 1064 nm wavelength, and volume depolarization ratio at 355 and 532 nm with a height resolution of 7.5 m and a temporal resolution of 30 s (for details see Engelmann *et al*.^63^). From these measurements, profiles of particle backscatter and particle linear polarization ratio can be derived. Additional detection of Raman-scattered light enables the independent determination of the particle extinction coefficient profiles at 355 and 532 nm and profiles of the water vapor mixing ratio during nighttime. The respective far-range channels reach a complete overlap in about 800 m and can receive signals up to 30 km height. To cover also the lower troposphere, four near-range channels with a complete overlap at 120 m height for the detection of elastic and inelastic scattering at 355, 387, 532, and 607 nm are implemented in the system. For the MOSAiC campaign, the lidar setup was extended with an additional near-range channel for polarization measurements at 532 nm. This channel is the basis of a dual field-of-view depolarization analysis to retrieve cloud droplet number concentrations^64^. From the lidar measurements, vertical profiles of aerosol optical properties can be retrieved^65^ which can be used for aerosol classification^66,67^, and to derive their particle size distribution and number concentration^68,69^. During low sunlight conditions, the water vapor mixing ratio can be retrieved by means of a Raman channel at 407 nm^70^. A shape classification, e.g., the separation between dust and non-dust in aerosol layers can be derived by using the depolarization channels^71^. Additionally, a cloud phase separation of hydrometeors can be retrieved using the depolarization signal^72^. This capability can be applied to study heterogeneous ice formation in mixed-phase clouds^26,39^ and to estimate CCN and INP concentrations^36,73^. From the relatively short wavelength of the lidar, it follows that the instrument is rather sensitive to small liquid droplets than larger ice crystals and attenuation from liquid clouds (and molecules) needs to be considered.

The MWR HATPRO measures the atmospheric emission in 7 channels between 22.24 and 31.4 GHz to retrieve the integrated water vapor (IWV) and LWP^61^. Using its scanning capabilities and the brightness temperature measurements at 7 channels between 51.0 and 58.0 GHz additionally temperature profiles of the planetary boundary layer can be determined. The respective MOSAiC dataset is described in detail in Walbröl *et al*.^74^. The column-integrated IWV and LWP are, e.g., used to constrain retrievals of cloud microphysical properties^75^ and they play an important role in determining the radiation budget at the surface^30^. For the Cloudnet dataset, only the LWP product from HATPRO was used, processed and published by the University of Cologne^76^.

The laser-optical disdrometer OTT Particle Size Velocity (Parsivel^2^) was used to estimate the precipitation rate. Parsivel^2^ provides the particle size distribution and fall velocity of hydrometeors passing the sampling area. The disdrometer can detect particle sizes between 0.2 and 25 mm and fall velocities between 0.2 and 20 m s^−1^ and based on which the type and rate of precipitation are derived. The Parsivel^2^ was located at the bow of Polarstern (see Fig. 2) where turbulence caused by the ship’s superstructure sometimes led to unrealistic precipitation amounts, similar to those reported in Matrosov *et al*.^77^.

#### ARM cloud radar KAZR

**Fig. 4:**
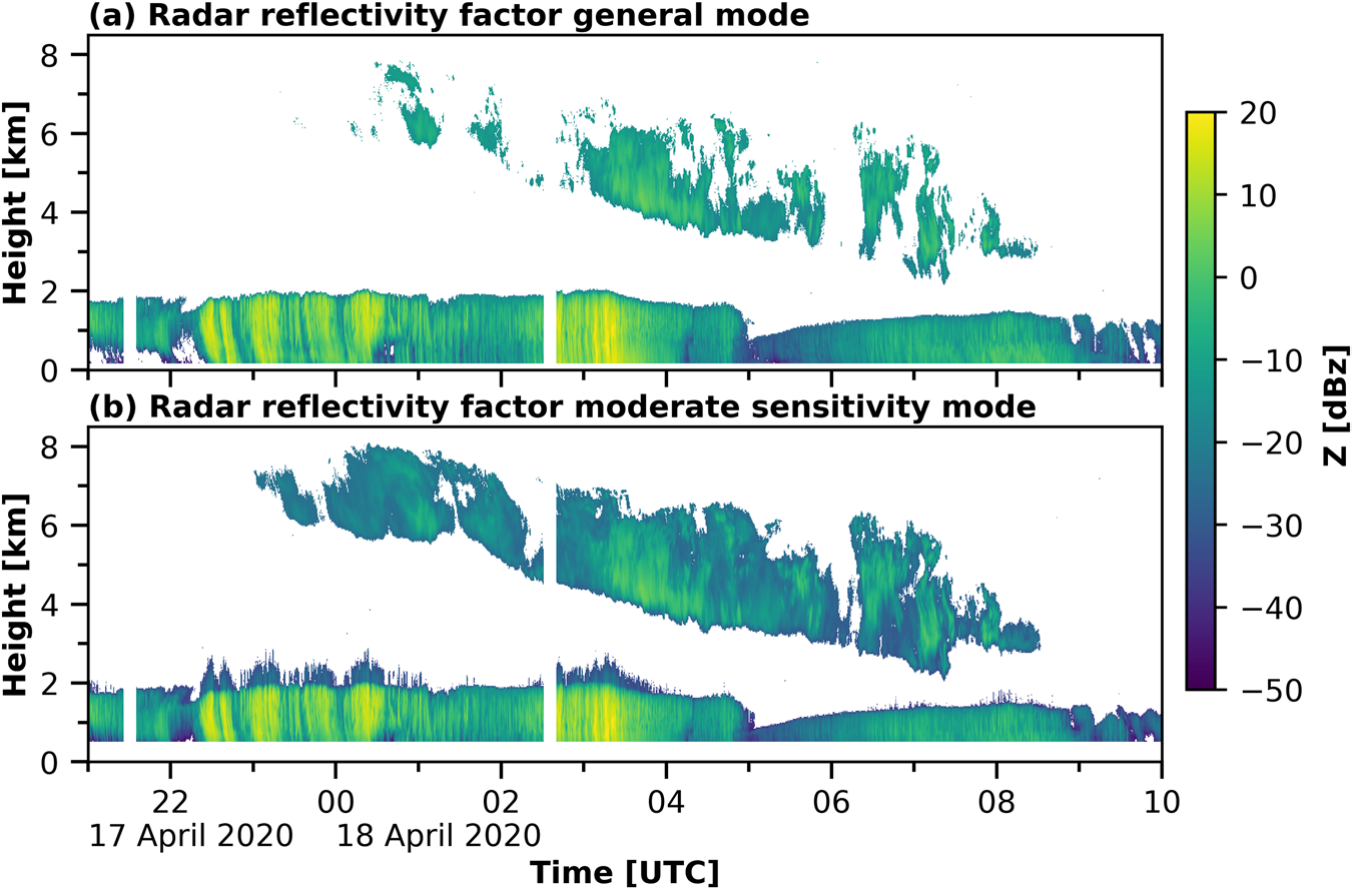
Cloud radar reflectivity factor from the KAZR GE mode (**a**) and MD mode (**b**) for 17 April 2020 21 UTC until 18 April 2020 10 UTC.

For micro- and macrophysical cloud observations, data from a zenith-pointing 35-GHz cloud radar Ka-band ARM Zenith Radar^78^ (KAZR) were used. The respective datasets were processed and published by ARM^79,80^. The KAZR provides profiles of Doppler spectra, of which the different Doppler moments such as radar reflectivity, Doppler velocity, and Doppler spectral width were determined with a temporal resolution of 2 s and a height resolution of 30 m. The larger wavelength applied by the cloud radar, in contrast to the lidar, defines its sensitivity to range from cloud hydrometeors to slight precipitation. Cloud radar measurements are, for example, applied in cloud phase separation approaches^81–83^ and retrievals of cloud microphysical properties^19,75,84–86^. KAZR was operated in two modes simultaneously during the MOSAiC expedition, the general (GE) mode and the moderate sensitivity (MD) mode. The GE mode applies a burst pulse, while the MD mode uses a frequency-modulated chirp pulse. By means of the frequency-modulated pulse, more energy is emitted compared to the burst pulse, which results in the higher sensitivity of the MD mode. The frequency modulation, however, produces range sidelobes, which interfere with the signal in the lowest range gates. Therefore, the MD mode is only available above 450 m. The burst pulse avoids such artifacts. Hence, the GE mode is already available at a height of 122 m above the radar. The lowest two range gates at 122 m and 152 m height, however, still suffer from overlap issues, and for the presented Cloudet dataset only KAZR data from 182 m altitude and above were used. Due to technical reasons, artifacts up to a height of 3 km were visible in the MD mode data. An example of these artefacts are shown in Fig. 4. Figure 4 (a) illustrates the cloud radar reflectivity between 17 April 2020 21:00 UTC and 18 April 2020 10:00 UTC from the GE mode is shown and in panel (b) from the MD mode. A stratus cloud was observed below 2 km height throughout the presented period, with a cirrus above. Frequently increased reflectivity just above the cloud top can be identified in the MD mode observations of the stratus cloud, which are not visible in the GE mode, e.g., between 17 April 2020 22:30 UTC and 18 April 2020 01:00 UTC and on 18 April 2020 between 02:30 and 04:00 UTC as well as around 08:00 UTC above 2 km. Simultaneously, the cirrus cloud is much better resolved in the MD mode observations, compared to the GE mode but did not show any artefacts. Hence, no MD mode data were used below an altitude of 3 km but above.

### Cloud macro- and microphysical properties: cloudnet

To derive continuous profiles of cloud macro- and microphysical properties, the instrument synergy approach of Cloudnet^18,48^ was applied to the introduced measurements. Initially, Cloudnet was developed as a MATLAB application to provide an instrument synergy approach to process remote-sensing data for model evaluation^18^. Recently, the Cloudnet code was transferred into a Python package called CloudnetPy^48^. Cloudnet provides its products, such as the target classification, the liquid and ice water content, and liquid droplet and ice crystal effective radius, on a time-height grid with a resolution of 30 s and approximately 30 m. Based on the Cloudnet products, for example, studies on the evaluation of cloud properties and their interaction with aerosol particles^19,84,87,88^, model evaluation^18,89,90^, and their radiative effect^28,30,91^ can be performed.

As an input, Cloudnet processes the lidar attenuated backscatter coefficient and depolarization ratio, the cloud radar reflectivity and Doppler velocity, the LWP from the MWR, the disdrometer rain rate, and the thermodynamic profile of the radiosondes. The high variability of Arctic clouds poses challenges to remote-sensing techniques, especially the high frequency of low-level clouds. To obtain the most comprehensive picture of the different cloud occurrences, the Cloudnet dataset was derived by combining the different assets provided by the single instruments, namely the Polly^XT^ near-range and far-range channels and the KAZR GE and MD modes. In the published dataset, the different observations were merged as follows: the lidar near-range data were used up to a height of 1 km. Above, the far-range data were utilized. The KAZR data were merged at a height of 3 km, since in the KAZR MD data artifacts up to a height of 3 km were found. Hence, a customized CloudnetPy version was applied, which was modified to handle the non-standard Cloudnet instrumentation operated during the MOSAiC expedition and the challenging conditions of Arctic clouds.

Based on the measurements, Cloudnet derives a bitmask, where for each pixel (i.e., data point) the following category is assigned as yes or no: ‘clear’; ‘liquid’; ‘falling’; ‘cold; ‘melting layer’; ‘aerosol’; ‘insects’^92^. The category assignment is decided based on different criteria. Liquid droplets are assumed to have a large effective surface area in the probed volume compared to ice crystals as they are in fact rather small but appear in high numbers. Their presence is determined by lidar measurements due to their sensitivity to small-sized but numerous particles. Hence, a Cloudnet pixel with high backscatter and a distinct attenuation of the signal is defined as liquid. For the liquid detection during the MOSAiC campaign the 532 nm channel was used. Therefore, as a first criterion for liquid cloud pixel identification, a peak attenuated backscatter coefficient *β* exceeding a threshold of 10^−5^ sr^−1^ m^−1^ was defined. As the second criteria for liquid detection, the derivative of *β* with height *h*

1 $$\frac{\Delta \beta }{\Delta h}=\frac{{\beta }_{{\rm{P}}}-{\beta }_{{\rm{T}}}}{{h}_{{\rm{T}}}-{h}_{{\rm{P}}}},$$

should exceed a threshold of 10^−7^ sr^−1^ m^−1^ m^−1^. The indices P and T represent the respective values at the peak maximum and peak top. The thresholds were derived empirically to ensure that the selected peaks have a well-defined shape and are part of a prominent liquid layer. A falling pixel was identified by a valid cloud radar signal and a cold pixel was determined by a wet bulb temperature *T* < 0 °C. To estimate the wet bulb temperature at the respective time-height pixel, radiosonde-based profiles of thermodynamic variables were interpolated on the Cloudnet grid. Both criteria, falling and cold were used for cloud ice identification. If the conditions of ice and liquid were fulfilled simultaneously within one Cloudnet pixel, this pixel was set as mixed-phase. To identify the melting layer, the wet bulb temperature, the cloud radar Doppler velocity *v* (downward directed Doppler velocities were defined as negative), and the cloud radar spectral width were used. The wet bulb temperature for melting layer detection was constrained between −4 and 3 °C. The cloud radar Doppler velocity *v* should be below −2 m s^−1^ at the melting layer base. In addition, a minimum increase in *v* between the melting layer base and melting layer top of more than 0.5 m s^−1^ was set for the melting layer detection. Finally, the spectral width should decrease by more than 0.2 m s^−1^ from the melting layer base to the melting layer top, and the vertical extent of the melting layer should be less than 1000 m. Due to their rather small size range but heterogeneous shape distribution, aerosol was characterized by the absence of a radar signal and low lidar backscatter signals which can show both strong or weak depolarization, depending on the aerosol type. The presence of insects was detected based on the heuristic probability of their occurrence. The probability was calculated based on the cloud radar reflectivity, Doppler velocity, and spectral width and the atmospheric temperature. Due to the high Arctic location, the probability-threshold for insect detection was set to 1. Aerosol particles were screened from the liquid and melting layers and were prohibited from being present at heights above the melting layers as a reliable aerosol layer detection above a melting layer is challenging because of the attenuation of the lidar signal due to the liquid water. During MOSAiC no situation of a melting layer with a lidar signal above, which would indicate aerosol particles, was observed.

**Fig. 5:**
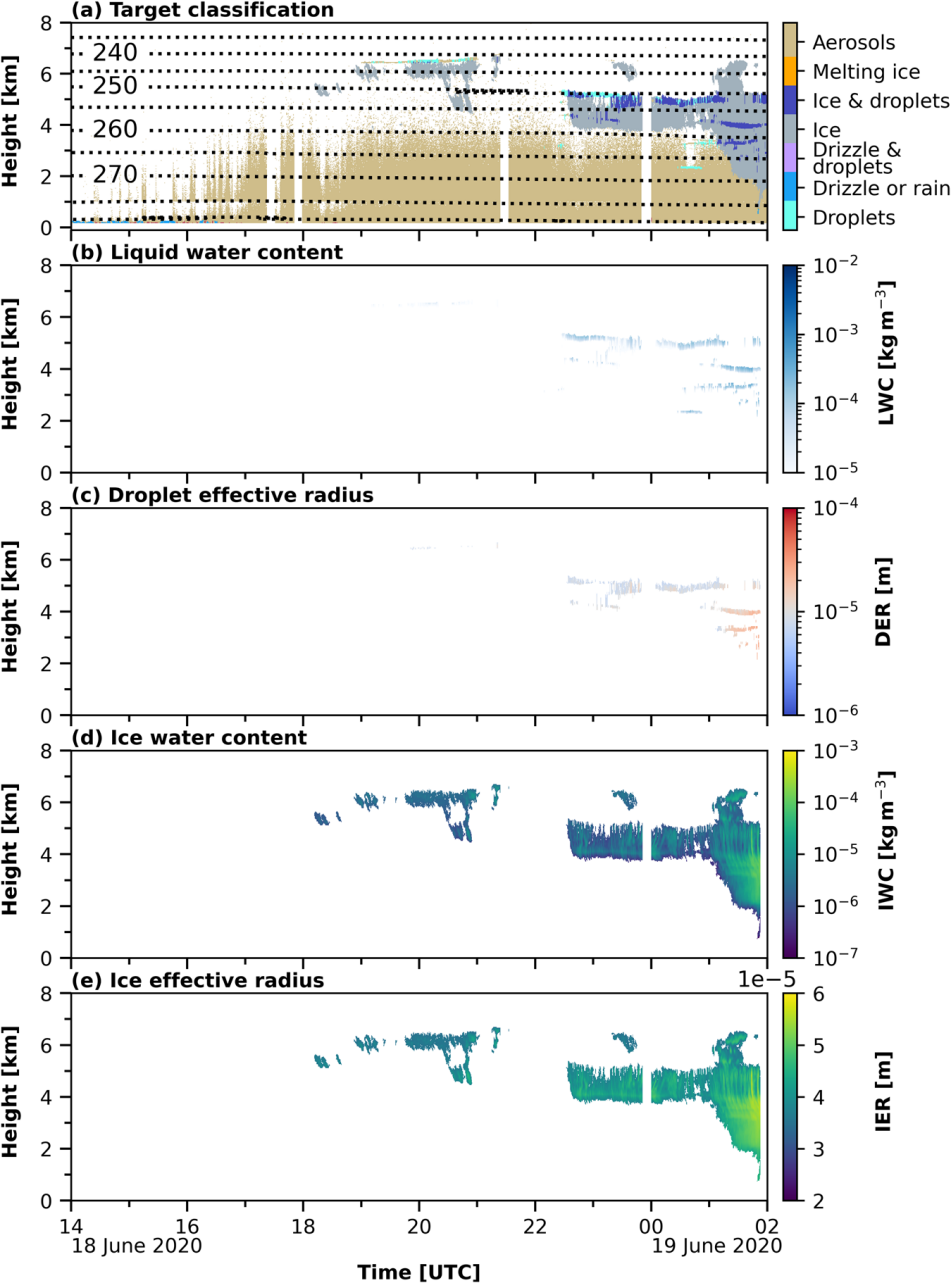
Derived Cloudnet products for the case study shown in Fig. 3. In panel (**a**) the Cloudnet target classification is shown together with the temperature [K] indicated by the dotted isotherms. Panel (**b**) and (**c**) present the liquid microphysical properties, namely the LWC and DER. The ice microphysical properties, IWC and IER are shown in panel (**d**) and (**e**), respectively.

Using the derived bit settings, each pixel was classified as ‘no data’, ‘aerosol & insects’, ‘insects’, ‘aerosol’, ‘melting & droplet’, ‘melting ice’, ‘ice & droplets’, ‘ice’, ‘drizzle & droplets’, ‘drizzle or rain’, or ‘droplets’. After the pixel classification, retrievals for the respective cloud microphysical properties were applied. The liquid water content (LWC) and liquid droplet effective radius (DER) were calculated for each pixel classified as liquid-containing (‘ice & droplets’, ‘drizzle & droplets’, or ‘droplets’) and for ice-containing clouds (‘ice & droplets’ or ‘ice’) alike. To derive the LWC, the LWP of the MWR was scaled adiabatically onto the liquid-cloud pixels. The theoretical adiabatic change of liquid water with height was calculated after Brenguier^93^ using the ambient pressure and temperature. The DER was calculated using the proposed method from Frisch *et al*.^85^ and was based on the cloud radar reflectivity. Both, the ice water content (IWC) and the ice crystal effective radius (IER) were calculated based on the cloud radar reflectivity and temperature using the approach from Hogan *et al*. for IWC and from Griesche *et al*.^19^ for IER. The specifications of the retrievals, the used input parameters, the uncertainty range, and the respective references are given in Table 2. Figure 5 depicts the derived Cloudnet products, i.e., the target classification (panel (a)), the LWC and DER (panel (b) and (c)), and the IWC and IER (panel (d) and (e)), for the measurements shown in Fig. 3.

**Table 2:** Cloudnet product specifications.

Product	Input parameter (Instrument)	Uncertainty	Reference
Liquid water content	LWP (MWR) Z_e_ (cloud radar) T, p (radiosonde) [*β* (lidar) for liquid identification]	±15% to ±25%	^75^
Liquid droplet effective radius	Z_e_ cloud radar [*β* (lidar) for liquid identification]	±15%	^85^
Ice water content	Z_e_ (cloud radar) T (radiosonde)	−30% to +40%	^86^
Ice crystal effective radius	Z_e_ (cloud radar) T (radiosonde)	±50%	^19^

#### Low-level stratus

The Arctic is known for its high occurrence of low-level clouds^31^, which are frequently located below the lowest detection limit of most remote-sensing instruments^19,94^. Yet, these clouds are often still too high for ground-based *in-situ* sensors. To fill the blind zone between the surface observations and the height of the first cloud radar pixel (182 m above the ground for this dataset), the LLS cloud detection approach from Griesche *et al*.^19^ was updated and applied to the MOSAiC dataset. These low-level clouds were identified by the lidar attenuated backscatter of the 532-nm near-field channel. If the attenuated backscatter exceeded a value of 4·10^−6^ sr^−1^ m^−1^ below the lowest Cloudnet range gate, a LLS cloud was classified. This threshold is based on the approach from Griesche *et al*.^19^ and was derived by sensitivity studies. The LLS occurrence for the case study presented in Fig. 3 is indicated by the purple flag shown in panel (e).

## Data Records

The Cloudnet data for the MOSAiC expedition were published via the ACTRIS Cloudnet data portal as daily netCDF files of the lidar observations, the Cloudnet categorize bitmask and the Cloudnet products, such as the target classification, DER, IER, IWC, and LWC^53–58^. The LLS dataset was published via Pangaea as daily netCDF files^95^. The issue dataset was published via Zenodo as daily netCDF files^96^. This issue dataset considers artefacts in the Cloudnet data, e.g., caused by tethered balloon or ship crane operations, which are introduced in detail in the next Section. This dataset might be updated in case new issues will be detected. The metadata of each dataset has been structured similarly based on the default of Cloudnet. For each file, additional information such as the source files, references, and contact are given in the global attributes. As the data was retrieved from observations made on a moving platform, the latitude and longitude information are stored as time dependent variables in the netCDF files. The different sets of data collections are summarized in Table 3.

**Table 3:** List of the different datasets, described in this document, their content, and references.

Product	Content	DOI & Reference
Categorize data	Observations Attenuation corrections (radar_liquid_atten, radar_gas_atten) Category bits Quality bits	21.12132/2.7d648da131c84518^53^
Target classification	Target classification bits Detection status Cloud base height Cloud top height	21.12132/2.eff342448cc74f7d^54^
LWC	Liquid water content Liquid water content retrieval status Random error in liquid water content	21.12132/2.f5846eca5ea84780^57^
DER	Droplet effective radius Cloud droplet number concentration Droplet effective radius (scaled to LWP) Absolute error in droplet effective radius Absolute error in droplet effective radius (scaled to LWP) Droplet effective radius retrieval status	21.12132/2.5aca23b91e064e44^55^
IWC	Ice water content Ice water content including rain Possible bias in ice water content Minimum detectable ice water content Random error in ice water content Ice water content retrieval status	21.12132/2.3c2801d5354344c9^57^
IER	Ice effective radius Ice effective radius including rain Ice effective radius retrieval status Random error in ice effective radius	21.12132/2.48e7517351394618^56^
LLS dataset	LLS flag LLS mask	10.1594/PANGAEA.961789^95^
Issue dataset	Issue bit Vessel tilt	10.5281/zenodo.731085^96^

## Technical Validation

To validate the dataset, first, a calibration of the cloud radar reflectivity is presented. The derived cloud boundaries were compared to the ARM KAZR Active Remote Sensing of Clouds (ARSCL^97^) MOSAiC dataset^98^. In the observational dataset, data issues caused by external drivers were identified, which are summarized subsequently.

### Radar calibration

**Fig. 6:**
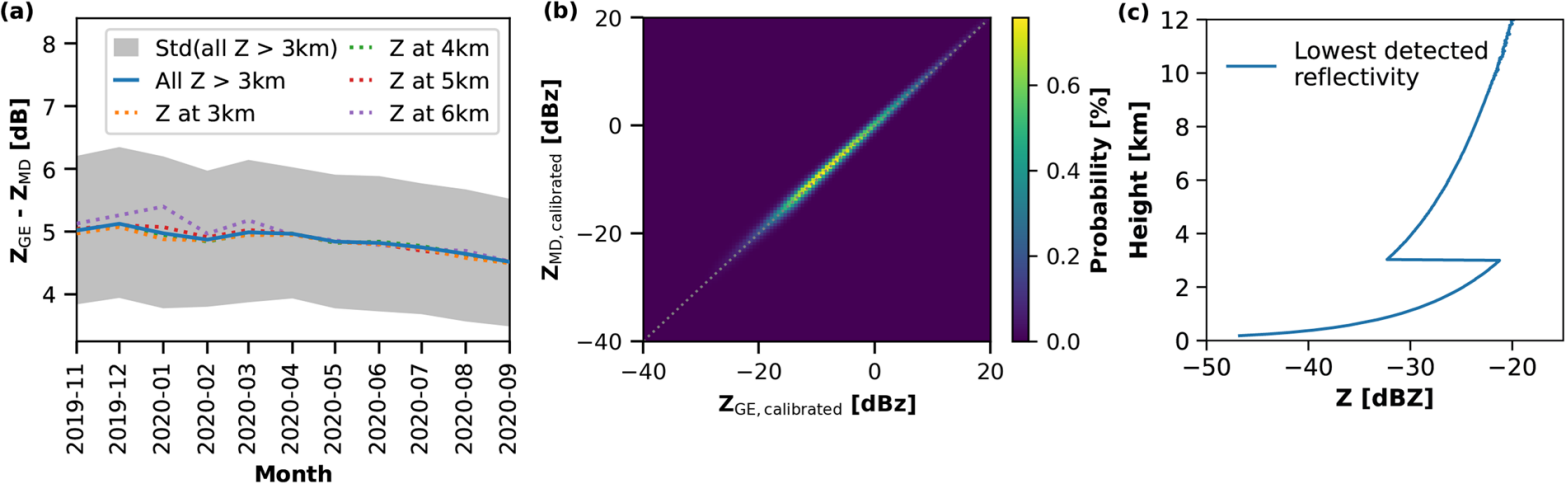
Panel (**a**) shows the Z_e_ offset between the KAZR GE mode and the KAZR MD mode. In blue the mean offset for all Z_e_ observed above a height of 3 km is shown. The gray shaded area depicts the standard deviation for all Z_e_ above 3 km height. The orange, green, red, and purple lines represent the reflectivities at different heights (3, 4, 5, and 6 km, respectively). In panel (**b**) the calibrated KAZR reflectivity of the general mode (GE) vs the moderate sensitivity mode (MD) during the MOSAiC campaign is shown. The dotted line indicates the 1-to-1 line. In panel (**c**) the lowest detected reflectivities during the MOSAiC campaign are shown for each range gate up to 12 km height.

The dependence of the retrievals for the cloud microphysical properties on the cloud radar reflectivity demands an accurate calibration of the latter. The usage of two different radar modes and hence two sets of cloud radar reflectivities needed a careful analysis of both datasets. Matrosov *et al*.^77^ evaluated the KAZR GE mode reflectivity Z_e_ derived during the MOSAiC expedition based on a comparison between the maximum reflectivity and the LWP^99^ and a comparison to the KAZR at the ARM site North Slope of Alaska (NSA, 71.325° N, 156.608° W). It was shown that the KAZR GE mode Z_e_ was 1 dB too low during the MOSAiC campaign. A direct intercomparison between Z_e_ from the GE mode and the MD mode is shown in Fig. 6. The median reflectivity differences between the two modes for all Z_e_ observed above 3 km, as well as for Z_e_ at different heights (3 km (orange), 4 km (green), 5 km (red), and 6 km (purple)) are shown. The average difference was around 5 dB. A slight decrease during the MOSAiC year can be seen, which was, however, below 0.5 dB. Finally, we have calibrated the KAZR GE Z_e_ data^79^ with + 1 dB as proposed by Matrosov *et al*.^77^ and have used this data in the Cloudnet processing up to a height of 3 km. Above 3 km height we used the KAZR MD Z_e_ data^79^, which was calibrated with + 6dB. After the calibration, the reflectivities of both, the GE and the MD modes showed a correlation of 0.97 (see Fig. 6b).

### Cloud boundaries

To validate the derived cloud properties, a comparison between the Cloudnet dataset and cloud boundaries from ARSCL was performed. ARSCL is a synergistic retrieval for remote-sensing to derive cloud macrophysical properties, based on cloud radar and lidar observations. The MOSAiC ARSCL dataset applied the same cloud radar observations as used in this Cloudnet dataset to derive the cloud top height but utilized different lidar systems for the determination of the liquid layer base. The lidar systems used in the ARSCL processing were a micropulse lidar (MPL)^100^ and a ceilometer^101^, which were part of the AMF2. The MPL and the ceilometer data were used to derive the cloud base height, and the cloud top height was estimated based on the cloud radar observations.

**Fig. 7:**
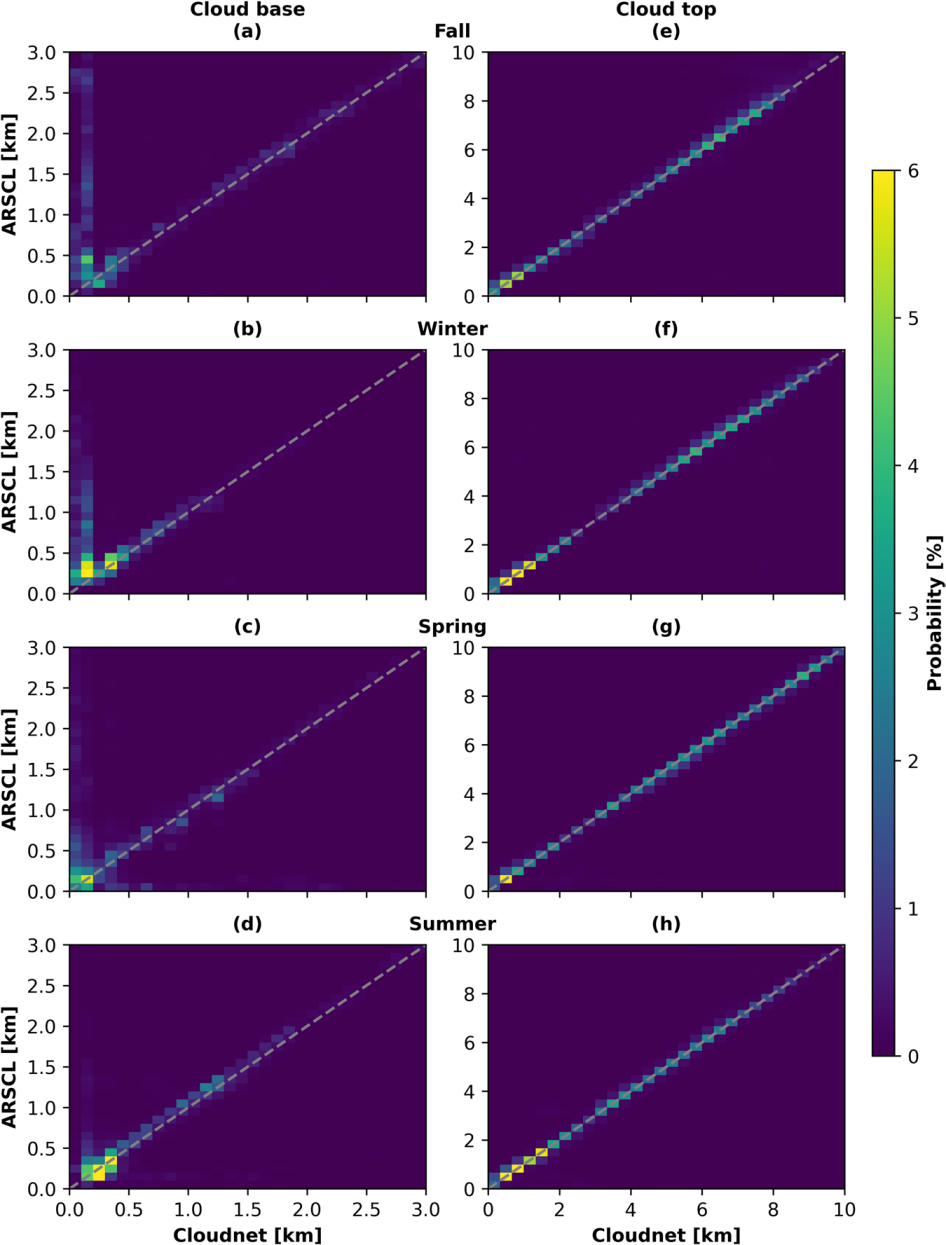
2D-histograms of the lowest liquid-dominated cloud layer base (left column) and cloud top heights (right column) from Cloudnet compared to ARSCL for fall, winter, spring, and summer. The dashed line in each panel indicates the 1-to-1 line.

In Fig. 7, the cloud top and the liquid layer base height from ARSCL and Cloudnet are contrasted against each other for fall (November 2019, September 2020, and October 2020), winter (December 2019, January + February 2020), spring (March – May 2020), and summer (June – August 2020). A very good correlation of the cloud top heights between both datasets is apparent in Fig. 7e–h. This agreement is also reflected in the R values, which were above 0.9 for every season. The liquid layer base heights derived from the two approaches scatter more, especially at lower altitudes. At higher altitudes, the values from both approaches follow the 1-to-1 line more closely. The R values for the liquid layer bases were 0.6, 0.59, 0.56, and 0.75 for fall, winter, spring, and summer, respectively. These differences were likely caused by the different lidar systems applied. In the case of the presented Cloudnet dataset a Polly^XT^ was utilized, while in the ARSCL data a combination of MPL and ceilometer was used. All three lidar systems differ in their sensitivities and lowest detection height, and therefore may detect the liquid base at different heights. The lowest detection height of the MPL is 150 m with a range resolution of 15 m^100^. For the ceilometer, a possible detectable cloud base height between 0–7500 m is specified with a vertical resolution of 10 m^101^. The Polly^XT^ is capable to detect clouds down to 50 m above the ground and has a vertical resolution of 7.5 m^63^. The ARSCL retrieval applies a backscatter together with an attenuation threshold for liquid cloud detection, similar to the liquid water detection scheme from Cloudnet. The LLS retrieval was developed without an attenuation threshold. This approach proved to be valuable to detect stratiform liquid-containing clouds as they can often be observed in the Arctic summer. A good performance of this approach for the summer 2017 was shown by Griesche *et al*.^19^ and can be seen by the good agreement between the ARSCL and Cloudnet cloud base shown in Fig. 7d.

**Fig. 8:**
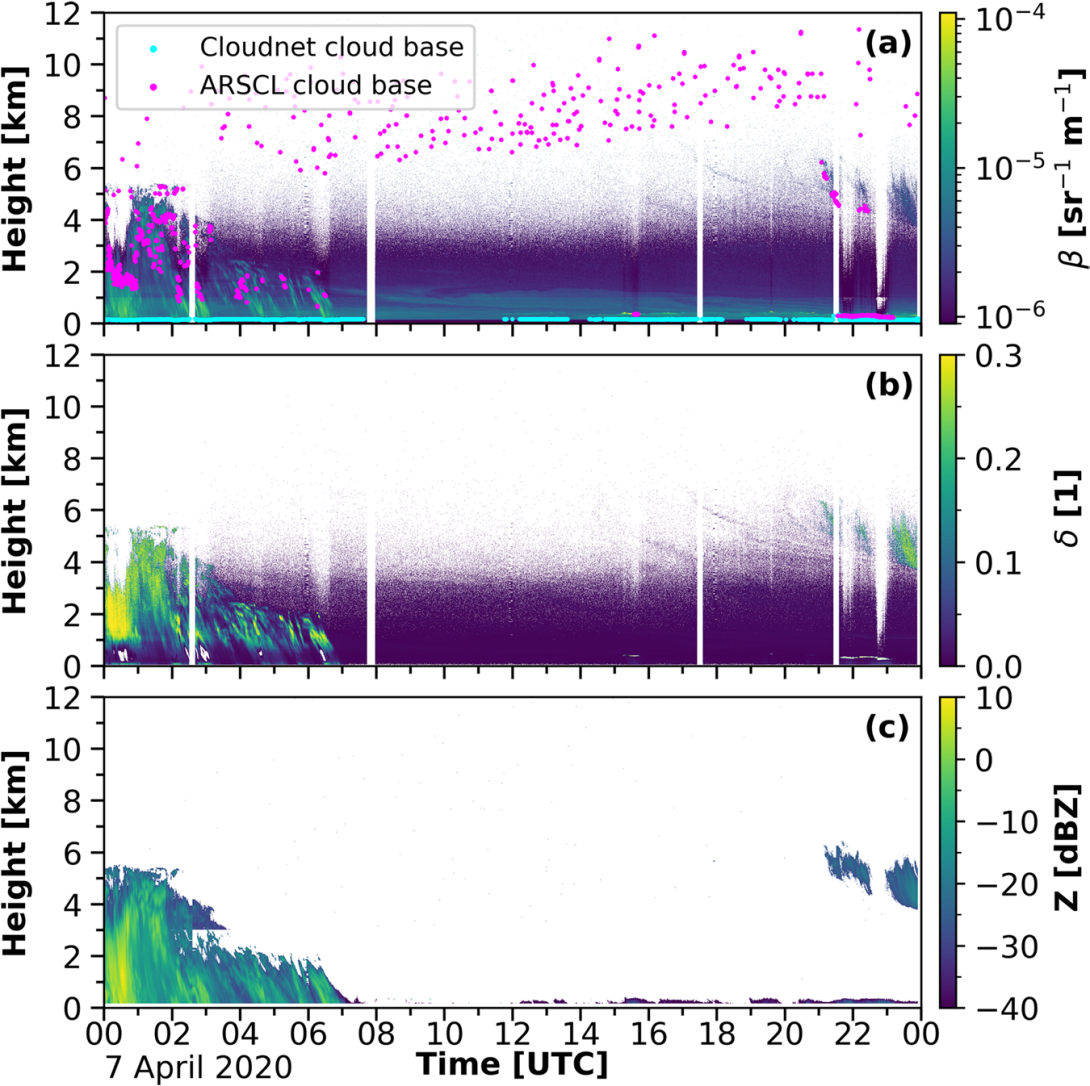
Evaluation of the cloud base detections by the LLS retrieval and ARSCL. Panel (**a**) shows the Polly^XT^ attenuated backscatter for 7 April 2020. The pink dots show the cloud base height as given in the ARSCL dataset and the blue dots the Cloudnet cloud base heights. In panel (**b**) and (**c**) the lidar volume depolarization and the cloud radar reflectivity factor are depicted, respectively.

The application of the backscatter-threshold LLS approach to the full MOSAiC dataset features a certain sensitivity to other atmospheric scatters, too, such as ice particles, precipitation, and humidified aerosol, which are located in the so-called cloud twilight zone^102^. An example for a low-level ice cloud detected by Cloudnet but not by ARSCL is shown in Fig. 8. Figure 8a shows the attenuated backscatter from Polly^XT^ for 7 April 2020 and the retrieved cloud base heights from Cloudnet (including LLS) in blue and ARSCL in pink. Figure 8b depicts the lidar volume depolarization and Fig. 8c the cloud radar reflectivity factor. A good agreement between both cloud base retrievals can be seen during the presence of the stratocumulus cloud between 21 and 23 UTC below 1 km height. During the rest of the day, Cloudnet frequently detects low-level clouds that did not cause strong attenuation and hence are not present in the ARSCL data. The tops of these low clouds are occasionally also indicated in the cloud radar observations, suggesting that these are likely ice clouds. Also, the base of precipitation features, as observed in the first 4 hours of the case study in Fig. 8, was detected. The ARSCL dataset, in turn, frequently detects high clouds above 6 km height, which do not correspond to the observations made by any of the available lidar systems or by the cloud radar. In total for the entire campaign, about 15% more low-level clouds were detected by Cloudnet than by ARSCL.

### Data issues

During the MOSAiC year some periods of corrupted observational data applied in the Cloudnet processing have been identified, namely in the lidar, cloud radar, and MWR measurements and were summarized as a bit flag in an additional issue dataset^96^. These issues were caused by external drivers and were documented in the ARM KAZR dataset publications^79,80^ (bits 1 & 2), or determined by tethered balloon operation periods (bit 3) and experienced-eye observations by the OCEANET staff (bits 4–6). The respective periods were not removed from the dataset but documented in the issue dataset. The documented data issues are as follows:

- Bit 1: The cloud radar time was off by 75 seconds. Not possible to verify consistency of the time shift (10 October 2019 00:00 UTC to 11 December 2019 20:45 UTC).
- Bit 2: Striations in cloud radar observations likely due to waveguide issue (10 October 2019 00:00 UTC to 27 October 2019 04:23 UTC).
- Bit 3: Artifacts in cloud radar observations due to tethered balloon operations.
- Bit 4: Artifacts in cloud radar observations due to crane operations above the instrument.
- Bit 5: Lidar signal attenuation due to blowing snow covering the lidar window.
- Bit 6: Artifacts in liquid water path (e.g., due to crane operations above the instrument).

**Fig. 9:**
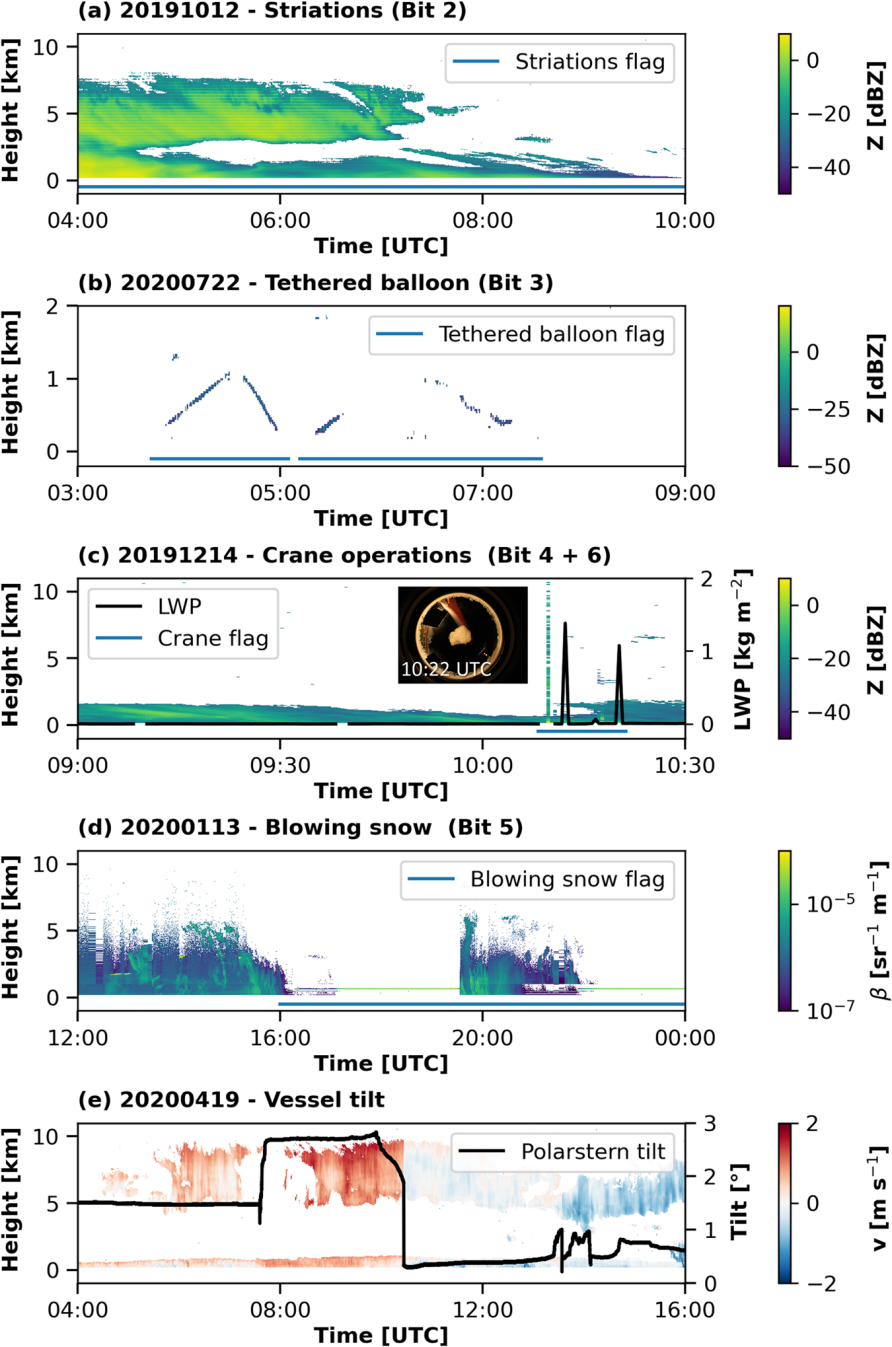
Examples for some of the issues identified during the processing of the MOSAiC Cloudnet dataset. Panel (**a**) shows the striations in the cloud radar observations. In panel (**b**) artifacts in the cloud radar reflectivity caused by two tethered balloon flights are depicted. Artifacts in the cloud radar reflectivity and LWP (black line) caused by crane operations are shown in panel (**c**). Panel (**d**) shows an example of blowing snow covering the lidar window. In panel (**e**) artifacts in the cloud radar Doppler velocity are shown together with the vessel tilt (black line). The blue lines in panels (**a**–**d**) indicate the flagged periods of the respective issue.

Additional biases in the cloud radar Doppler velocity caused by a non-leveled orientation of the research vessel and hence an off-zenith pointing of the cloud radar were occasionally identified (see Fig. 9e). For the vessel-tilt, no dedicated bit was defined, but continuous tilt values published within the issue dataset, to allow the user an own estimation of the reliability of the Doppler velocity.

In Fig. 9, some of the identified issues are exemplified. Figure 9a depicts the cloud radar reflectivity on 12 October 2019 between 04:00 and 10:00 UTC and striations were present throughout the whole presented period (bit 2). Figure 9b shows the cloud radar reflectivity on 22 July 2020 between 03:00 and 09:00 UTC. Between 03:44 and 07:35 UTC clutter from the tethered balloon is apparent, despite an actual clear sky situation (bit 3). In Fig. 9c the cloud radar reflectivity and MWR LWP are shown for 14 December 2020, together with a picture of the crane taken from the all-sky camera at 10:22 UTC. Between 10:08 and 10:22 UTC artifacts caused by crane operations can be seen in the reflectivity (bit 4) and LWP (bit 6). In Fig. 9d the lidar attenuated backscatter for 13 January 2020 between 12:00 and 14 January 00:00 UTC is shown. Blowing snow covering the lidar window and causing a lidar signal attenuation is obvious after 16:00 UTC (bit 5). Figure 9e depicts the cloud radar Doppler velocity for 19 April 2020 between 04:00 and 16:00 UTC together with the tilt values, shown as the black line. Dubious strong updrafts throughout the whole column, up to 2 m s^−1^ in the cirrus cloud until 10:26 UTC together with tilt values up to 3° were observed. After a realignment of the vessel at 10:27 UTC the cloud radar Doppler velocity was more centered at approximately 0 m s^−1^.

## Usage Notes

To introduce the usage of the dataset, two case studies, one from January and one from June 2020 are discussed here. The first case study will introduce the different data products provided in this dataset and their application for radiative transfer simulations. The second case will present a comparison of the LWC and IWC between Cloudnet and ECMWF Reanalysis v5 (ERA5)^103,104^.

### Case study 18 – 19 June 2020 - Observations, derived products, and application example

To introduce the usage of the dataset, a case study between 18 June 2020 14 UTC and 19 June 2020 02:00 UTC is discussed here. In Fig. 3, the observations, and in Fig. 5, the derived Cloudnet products are depicted. Figure 3a,b show the cloud radar reflectivity and Doppler velocity, which were used to identify ice clouds. In Fig. 3c,d, the lidar attenuated backscatter and volume depolarization ratio are presented. The lidar data were utilized for the liquid classification. Figure 3e shows the MWR LWP, which was used to constrain the LWC, and the LLS flag. The presented case started with a period of LLS. These low-level clouds attenuated the lidar signal below the lowest Cloudnet range gate, such that no microphysical properties were derived until 18 June 2020, 18:00 UTC, even though LWP up to 0.45 kg m^−2^ was measured by HATPRO. Between 18:00 and 21:30 UTC a liquid-layer topped ice cloud at 6 km height was observed over Polarstern, with ice precipitating from the liquid layer at 6.5 km height and at temperatures below −30 °C. After 22:30 UTC, an altocumulus cloud, again, with a liquid layer at cloud top at 5 km height and ice precipitation below was present. Until 19 June 2020 01:00 UTC, the ice sublimated at 4 km height. Subsequently, the ice crystals fall into lower liquid layers present at 4 km and 3.5 km altitude, probably causing riming. Hence, precipitation was observed down to a height of 1 km.

**Fig. 10:**
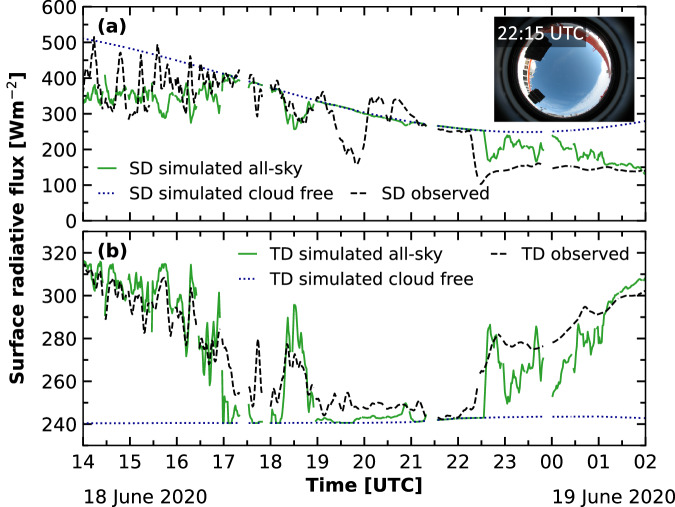
Time series of simulated all-sky (solid, green) and cloud free (dotted, blue), and observed (dashed, black) radiative downward surface fluxes for the case study introduced in Fig. 3. Panel (**a**) shows the downward solar radiative fluxes (SD) and in panel (**b**) the downward terrestrial radiative fluxes are depicted. The simulations are based on the cloud properties from Cloudnet shown in Fig. 5. Gaps in the time series are due to missing input data and a 5 minute running mean was applied to the data for clarity reasons.

Based on these observations and the derived cloud properties, for example, radiative transfer simulations can be done. To illustrate the use of the Cloudnet retrievals and the identification of LLS clouds for radiative studies, radiative transfer simulations with the TROPOS Cloud and Aerosol Radiative Effect Simulator (T-CARS)^28,105,106^ were performed for the case study presented in Figs. 3, 5. The T-CARS environment applies the single column 1D Rapid Radiative Transfer Model for General Circulation application (RRTMG)^107–109^. The simulations were conducted following the approach presented in Griesche *et al*.^30^ to include also the effect of the LLS, for which no liquid properties were derived by Cloudnet by default. The applied method used the LLS flag to identify the low-level clouds and compared the LWP measured by the MWR HATPRO with the column-integrated LWC. In case both values differed, the LWP was scaled adiabatically to the LLS layer and the DER was determined using a linear relationship between LWP and DER following Griesche *et al*.^30^. The simulation including the influence of clouds is shown in Fig. 10, together with the surface observations made aboard Polarstern and the simulation assuming a cloud-free situation. In general, a good agreement between the observations and the simulation was achieved in the downward surface solar (SD) and terrestrial (TD) radiative fluxes. However, some features were not reproduced by the 1-D simulations, such as 3-D effects from broken clouds, visible in the SD fluxes that exceed the clear-sky simulations (e.g., on 18 June 2020 20:00–21:00 UTC). Additionally, the effects of clouds appearing in the vicinity were not entirely captured by the simulations, as these clouds were not or not yet within the zenith-looking field of view of the remote-sensing instruments (as illustrated by the small all-sky camera picture in the upper right of Fig. 10a). Yet, due to the low sun-elevation angle, these clouds can already affect the incoming radiation and their influence is then visible in the radiative flux measurements. Effects such as these caused the overestimation of the simulated SD radiative fluxes of up to 150 W m^−2^ on 18 June 2020 from 19:00–20:00 UTC and after 22:00 UTC. Consequently, the differences were smaller in the TD radiative fluxes (below 40 W m^−2^). Overall, the mean differences between the simulations and the observations in the presented period were 9.22 W m^−2^ for SD and −3.60 W m^−2^ for TD.

### Case study 28 – 29 January 2020 - Reanalysis comparison

**Fig. 11:**
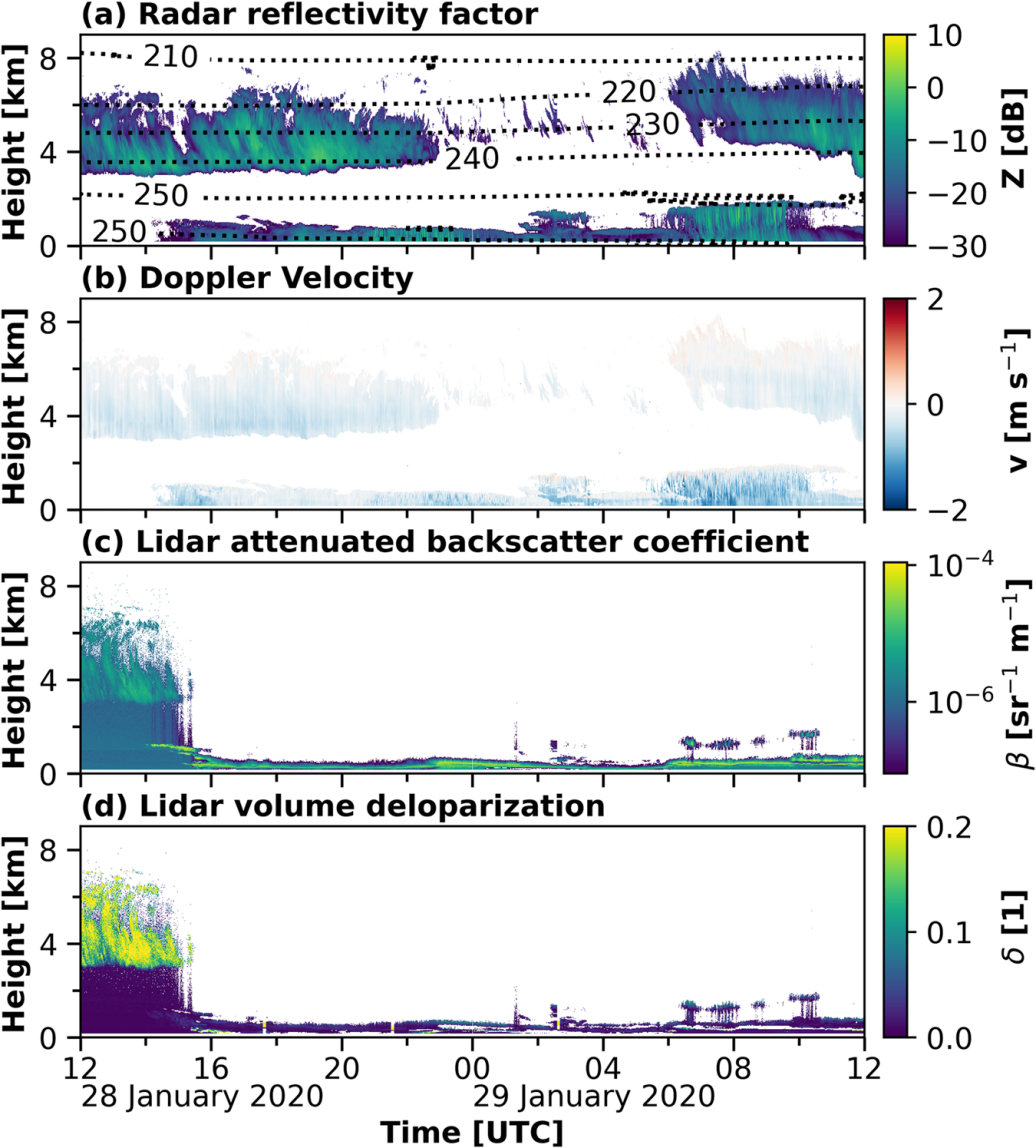
Observations from 28 January 2020 12 UTC until 29 January 2020 12 UTC between 0 and 9 km height as used in the Cloudnet processing. Panel (**a**) shows the cloud radar reflectivity together with the isotherms [K] from the radiosonde launches. In panel (**b**), (**c**), and (**d**) the cloud radar Doppler velocity, lidar attenuated backscatter, and lidar volume depolarization are depicted, respectively.

**Fig. 12:**
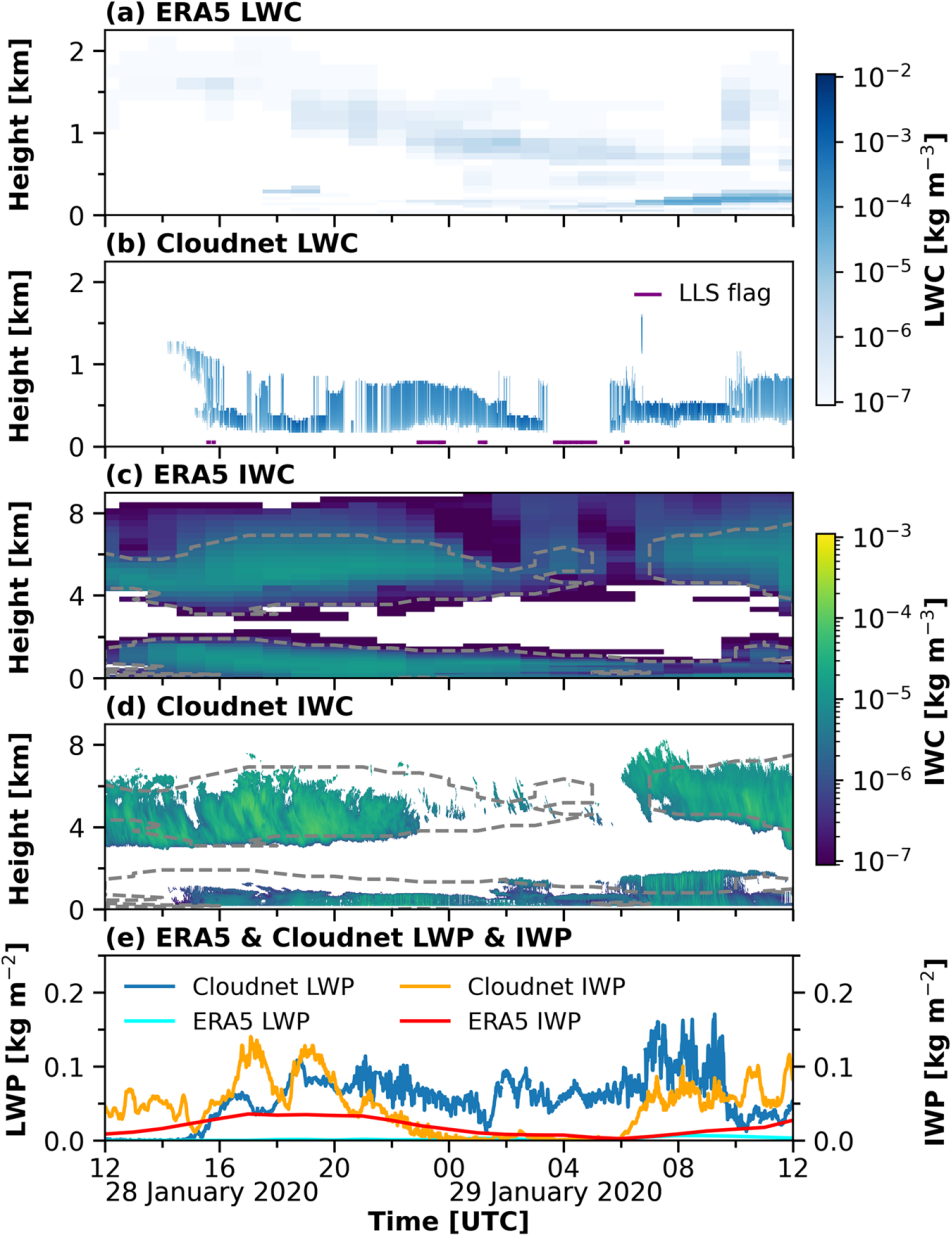
Case study of the application of Cloudnet for model evaluation. Panel (**a**) and (**b**) show the LWC and panel (**c**) and (**d**) show the IWC from ERA5 and Cloudnet, respectively, for the case presented in Fig. 11. The dashed lines in panel (**c**) and (**d**) highlight the respective IWC sensitivity of the cloud radar. In panel (**e**) the respective LWP and IWP are depicted.

Figure 11 shows the observations made from 28 January 2020 12:00 UTC until 29 January 2020 12:00 UTC. This case study was chosen to highlight the possibility of the dataset to tackle remaining challenges in the retrieval of height-resolved cloud parameters, from both, observations and modelling. A cirrus cloud between 3 and 7 km height was visible in the cloud radar reflectivity and Doppler velocity shown in Fig. 11a,b, respectively, throughout most of the presented period, with a discontinuity from 28 January 2020 23:00 UTC until 29 January 2020 05:00 UTC. This cloud likely only consisted of ice particles, as can be see from the low lidar attenuated backscatter coefficient in Fig. 11c together with the high lidar volume depolarization in Fig. 11d, and an LWP equal to zero (Fig. 12e) until 28 January 2020 15:00 UTC. The formation of the ice particles was mostly initiated at temperatures below the homogeneous freezing temperature of −38 °C as shown by the isotherms in Fig. 11a. After 28 January 2020 15:00 UTC a stratus cloud below 2 km height prevented further lidar analysis of the cirrus cloud, as it caused a complete attenuation of the lidar signal. The observed LWP from HATPRO for this mixed-phase cloud was between 50 and 170 g m^−2^ (Fig. 12e). The derived cloud properties from Cloudnet were compared to results from ERA5. ERA5 is a global reanalysis with assimilated observations to derive the best estimate of the atmospheric state on a horizontal grid with 31 km resolution and 137 vertical model levels from the surface up to 80 km altitude. For comparison with the Cloudnet properties the closest ERA5 grid point to the observations (latitude 87.3, longitude 96.0) was chosen. In Fig. 12 the LWC, IWC, LWP, and IWC derived form Cloudnet are compared to the same quantities from ERA5 for the period shown in Fig. 11. The Cloudnet IWP and the ERA5 LWP and IWP were derived as the vertical integral of the respective LWC and IWC. For a period of two hours on 29 January 2020 between 03:30 UTC and 05:30 UTC the stratus cloud caused an attenuation of the lidar signal already below the lowest cloud radar range gate, as indicated by the LLS flag in Fig. 12b. Hence, no LWC was derived by Cloudnet during this time period. The LWC derived from the observations ranged between 10^−4^ and 10^−2^ kg m^−3^, while the modeled LWC was mostly between 10^−7^ and 10^−4^ kg m^−3^. These differences in the LWC are reflected in the LWP, which is much smaller in the ERA5 simulations compared to the observations, a feature that was already reported in earlier studies^110^. Additionally, the location of the liquid differs between the observations and the simulations. Cloudnet identified for most of the time the liquid cloud between 150 and 1000 m height. In the ERA5 model results, two less pronounced layers were modeled. One layer which descended from between 1500 and 2000 m down to below 1000 m and another one that appeared at 22 UTC below 500 m. The lower cloud contained most of the modeled liquid water. After 29 January 2020 06:00 UTC, this cloud was located below the liquid cloud identified by Cloudnet. The location and magnitude of the ice water content matches between Cloudnet and ERA5. The gap in the cirrus cloud is less pronounced in the modeled ice cloud and the stratus cloud appeared earlier in the model than in the observations. A reason for differences in the modeled and observed IWC is due to different sensitivities of the model and the cloud radar. The model can simulate IWC values, which are not detectable by the cloud radar. The lowest reflectivity detected by the cloud radar for each range gate up to 12 km height is shown in Fig. 6c. The jump in the lowest detected reflectivity at 3 km height is due to the switch from the GE mode to the MD mode data. The ERA5 IWC was converted to cloud radar reflectivity, using the dependency presented in Hogan *et al*.^86^. The dashed lines in Fig. 12c,d highlight the regions where the modeled IWC was large enough to be detectable by the cloud radar. Considering these different sensitivities, we find a very good agreement of the spatiotemporal distribution of the ice location during the presented case between the model and the observations. The IWP from Cloudnet and ERA5 are overall consistent and in the range of previously reported differences between the model and observations^111^.

As presented in the two case studies, the introduced dataset can be used in radiative transfer simulations^112^, model evaluation, and cloud studies. It has already been applied as a reference for tethered balloon studies^113,114^, as ground-truth for a modeling study on a warm-air intrusion during the MOSAiC cruise^115^, and for a study on the influence of sea-ice leads on cloud properties^116^.

## Data Availability

The presented dataset was processed based on CloudnetPy version 1.39.0. However, to account for the Arctic clouds, modifications such as merging the observational data and the LLS processing needed to be done. The adjusted Cloudnet source code is archived via Zenodo^117^.
